# Meiotic Cytokinesis in *Saccharomyces cerevisiae*: Spores That Just Need Closure

**DOI:** 10.3390/jof10020132

**Published:** 2024-02-06

**Authors:** Matthew Durant, Xheni Mucelli, Linda S. Huang

**Affiliations:** Department of Biology, University of Massachusetts Boston, Boston, MA 02125, USA; matthew.durant001@umb.edu (M.D.); xheni.mucelli001@umb.edu (X.M.)

**Keywords:** meiosis, cell cycle, budding yeast, prospore membrane, cellularization, meiotic exit

## Abstract

In the budding yeast *Saccharomyces cerevisiae*, sporulation occurs during starvation of a diploid cell and results in the formation of four haploid spores forming within the mother cell ascus. Meiosis divides the genetic material that is encapsulated by the prospore membrane that grows to surround the haploid nuclei; this membrane will eventually become the plasma membrane of the haploid spore. Cellularization of the spores occurs when the prospore membrane closes to capture the haploid nucleus along with some cytoplasmic material from the mother cell, and thus, closure of the prospore membrane is the meiotic cytokinetic event. This cytokinetic event involves the removal of the leading-edge protein complex, a complex of proteins that localizes to the leading edge of the growing prospore membrane. The development and closure of the prospore membrane must be coordinated with other meiotic exit events such as spindle disassembly. Timing of the closure of the prospore membrane depends on the meiotic exit pathway, which utilizes Cdc15, a Hippo-like kinase, and Sps1, an STE20 family GCKIII kinase, acting in parallel to the E3 ligase Ama1-APC/C. This review describes the sporulation process and focuses on the development of the prospore membrane and the regulation of prospore membrane closure.

## 1. Introduction

Meiosis is a specialized form of cell division that results in the formation of haploid gametes used for sexual reproduction. In the budding yeast *Saccharomyces cerevisiae*, meiosis occurs as cells undergo sporulation, which happens when diploid cells are starved of nitrogen and a fermentable carbon source. Four haploid spores are formed within the diploid mother cell upon completion of sporulation; the plasma membrane and cell wall from the former mother cell become the ascus that encases the four spores.

**Figure 1:**
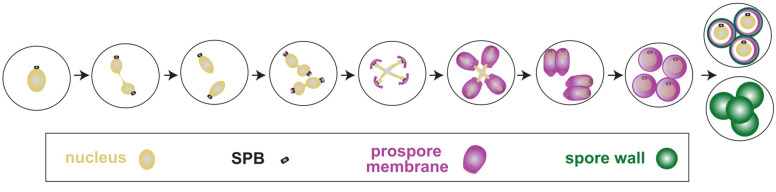
Meiosis occurs during sporulation in *S. cerevisiae*. Cartoon depicting sporulation in *Saccharomyces cerevisiae*. Progression through sporulation is diagrammed from left to right. Nuclei are labelled in yellow, spindle pole bodies in black, and prospore membranes in magenta. The mannan, β-glucan, chitosan, and dityrosine layers of the spore wall are labelled pink, turquoise, dark blue, and green, respectively.

Sporulation involves both the processes of meiosis and spore morphogenesis (Figure 1). Meiosis in *S. cerevisiae* occurs within the cytoplasm of the mother cell. The formation of the spores within the mother cell requires the de novo generation of two distinct cellular structures: a new phospholipid membrane for each daughter cell called the prospore membrane, and a new cell wall that contains different components from the vegetative yeast cell wall called the spore wall. Cellularization of the spore occurs when the prospore membrane closes; this is the cytokinetic event of meiosis in budding yeast. The sporulation process was reviewed extensively in [1]. The chromosomal duplication, recombination, and segregation aspects of meiosis, including meiosis I and the transition to meiosis II, have been recently reviewed [2]. Here, we focus on more recently published work related to meiosis II and meiotic cytokinesis in *S. cerevisiae*.

## 2. Sporulation in *S. cerevisiae* Involves Meiosis and Spore Morphogenesis

During sporulation, cells undergo both meiosis and spore morphogenesis. The early phase of sporulation is marked by gene expression shifts from mitotic genes to early meiotic genes, which begin when diploid cells lack nitrogen and lack a fermentable carbon source (reviewed in [3]). During starvation, diploid cells will alter gene expression profiles as they exit the mitotic cell cycle in G1 and enter the premeiotic S phase in preparation for the events of meiosis, including recombination, homology pairing, and two rounds of chromosomal segregation (reviewed in [2]).

Spore morphogenesis begins at meiosis II, with the modification of the cytoplasmic face of the spindle pole body (SPB) with a protein coat called the meiotic outer plaque, which will serve as membrane nucleation sites for the initiation of the prospore membrane [4,5,6]. The SPB is the yeast equivalent of the centrosome, and functions as the sole microtubule-organizing center in *S. cerevisiae* cells. In meiosis, the SPBs duplicate both at the beginning of meiosis I and again at the beginning of meiosis II, resulting in the four SPBs required for the second nuclear division. During meiosis II, the four SPBs change composition and function both in microtubule nucleation and membrane nucleation [4]. The modified SPBs are expanded and replace the microtubule nucleator Spc72 with a group of proteins that form the meiotic outer plaque. The meiotic outer plaque consists of Spo21 (also called Mpc70), Mpc54, Spo74, and Ady4 [7,8,9]. The deposition of Spo21 and Spo74 depend on Pfs1 (also called Ady1) [10,11]. Spo21 and Mpc54 are both coiled-coil proteins that have their N-termini facing the cytoplasmic face of the meiotic outer plaque [12]. The C-termini of both Spo21 and Mpc54 face the N-terminus of Cnm67, a constitutive SPB member [13]. 

As cells undergo meiosis, the number of nuclei that are packaged into spores can vary from one (monad) to up to four (tetrad). The reasons for packaging fewer than the expected four spores can vary. Disruption of the meiotic division process can lead to a situation where two diploid spores are packaged, as seen in *spo12*∆ and *spo13*∆ mutants [14]. Sometimes, cells package fewer than four spores, even when the meiotic divisions occur. The situations where two haploid spores are packaged (forming dyads) can be categorized into two genetically distinct types: those that package nonsister haploid spores [11,15,16,17] versus those that package spores randomly (such as in *ady3*∆ mutants [18,19]).

There are at least two distinct mechanisms that cells use to package fewer than four haploid spores. The process by which cells package nonsister dyads (as well as triads of three spores and monads of one spore) has been termed spore number control, and depends on the availability of acetate [15,16]. Cells may choose to package fewer spores if the nutritional environment is unfavorable; this process depends on the Ras/cAMP/Protein Kinase A pathway [11]. Spore number control occurs by modifying fewer SPBs, which leads to the formation of fewer prospore membranes and thus fewer spores. This is in contrast to the random packaging of spores, as described in *ady3*∆ mutants, which utilizes a different (and not yet fully understood) mechanism: *ady3*∆ cells can produce four prospore membranes but will often have fewer than four spores reaching maturity [18,19].

Sporulating cells modify their SPBs depending on the nutrients present in the environment. An intermediate metabolite in the glyoxylate pathway is required for modification of all four SPBs [15]. In the absence of this metabolite, cells preferentially modify the two daughter SPBs formed in the transition from meiosis I to meiosis II, leading to the packaging of only two spores and the formation of dyads (two spores packaged within a mother cell ascus) [15].

The production of the components of the meiotic outer plaque (Mpc54, Spo74, and Mpc70) relies on the concentration of acetate in the media of sporulating cells [16]. The levels of meiotic outer plaque components directly correlate to sporulation efficiency: lower levels of meiotic outer plaque protein expression lead to fewer SPB modifications, ultimately resulting in fewer than four spores [16]. A core component of the mitotic exit network (the NDR/LATS kinase complex Mob1-Dbf2 [20]) is repurposed for spore number control in meiosis to modify the SPB components [17,21].

**Figure 2:**
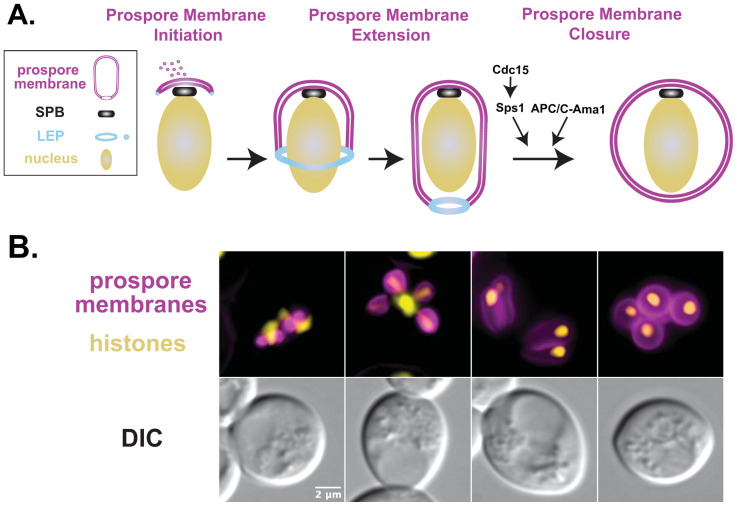
Prospore membranes undergo various morphological changes during meiosis II. (**A**) Cartoon depicting the stages of a single growing prospore membrane during sporulation. The prospore membrane is labelled magenta, nucleus in yellow, meiotic outer plaque in black, and leading-edge protein complex (LEP) in blue. Pathways controlling closure of the prospore membrane are indicated. Details in text. (**B**) Prospore membrane development in wild-type SK1 cells (LH1146), which contain the genomically integrated prospore membrane marker E20 (*his3::SPO20^51–91^-GFP^ENVY^:HIS3* [25,26]) shown in magenta and histones (*HTB2-mCherry:TRP1* [27]) shown in yellow. Scale bar = 2 microns. Fluorescent images are maximum intensity projections of 3 µm z-stacks merges taken on a Zeiss Axioskop Mot2 wide-field microscope, using a 100× objective (NA 1.45).

During spore morphogenesis, the prospore membrane grows through the fusion of post-Golgi vesicles at the meiotic outer plaque to surround the new meiotic products (Figure 2) [5,6]. Lipid droplets directly contact the prospore membrane in meiosis II and may also contribute to the expansion of the prospore membrane [22]. The four prospore membranes grow as double lipid bilayers to encapsulate each haploid nucleus, rounding up and closing at the end of meiosis. The outer bilayer lyses during spore wall deposition while the inner bilayer becomes the plasma membrane of the newly formed spore [23]. Closure of the prospore membrane leads to cellularization and is the cytokinetic event of meiosis, as this event separates the nuclei of the daughter cells from the mother cell cytoplasm and also captures some cytoplasmic components from the mother cell. Aging-related factors are kept in the mother cell and segregated away from the spore during sporulation, creating robust gametes (reviewed in [24]).

The prospore membrane is important for spore morphogenesis, as the spore wall is deposited within the lumen of the double lipid bilayer of the prospore membrane. The spore wall differs from the vegetative cell wall and is important for the ability of the spore to survive harsh environmental conditions [28]. Spore wall deposition utilizes the prospore membrane as a template and is an important part of spore maturation and viability [23,29]. The spore wall is made up of four distinct layers: two inner layers made up of mannan and β-glucan, and two outer layers made of chitosan and dityrosine (reviewed in [1,30]). The mannan layer is first deposited into the lumen of the prospore membrane, followed by the glucan layer [31]. Following deposition of the inner spore wall layers, the outer lipid bilayer of the prospore membrane lyses [23]. The deposition of the glucan layer is negatively regulated by the sporulation-specific MAP kinase *SMK1*, which is needed for cells to transition from the deposition of the inner spore wall to the deposition of the outer chitosan layer [32]. The chitosan layer is needed so that the dityrosine layer can be properly deposited [23]. The formation of the outer spore wall layers requires lipid droplets, which have been proposed to activate chitin synthesis in a manner that requires *SRT1* [22,33,34]. During spore maturation, spores form interspore bridges that are continuous with the outer layers of the spore wall; these bridges hold spores together when the ascal wall is disrupted [23]. Spore maturation also involves the rupture of the mother cell vacuole, leading to digestion of incompletely developed spores and compaction of the ascus [35,36].

The spore wall is distinct from the cell wall of vegetatively growing cells, which are composed of an inner layer of chitin and β-glucan and an outer mannan layer; this order is different from the spore wall whose innermost layer is the mannan layer (reviewed in [30,37,38,39]). Furthermore, the two outer layers (chitosan and dityrosine) are not present in the vegetative cell wall and allow the spore to survive harsher conditions (including the *Drosophila* intestinal tract [40]) compared to a vegetatively growing cell [29,41,42].

Autophagy is also essential for sporulating cells (reviewed in [43]), as cells lacking important components of the autophagic pathways do not form spores [44,45,46,47,48,49]. Autophagy is required for the clearance of amyloid-like Rim4 aggregates, which prevent translation of middle and late meiotic transcripts [50,51,52,53]. In addition to the role of Rim4 in mRNA sequestration and regulation, Rim4 also stimulates autophagy through activation of the Atg1 kinase [54,55]. Autophagy is needed for progression through meiosis through the degradation of cyclins and the polo-like kinase Cdc5 as well as the release of the phosphatase Cdc14 from the nucleolus during anaphase II [50]. Interestingly, Cdc14 also appears to promote autophagy during sporulation through the dephosphorylation of Atg13 [56] and by regulating the localization and autophagic destruction of Rim4 [55]. Thus, autophagy is an important regulator of meiotic progression.

## 3. Sporulation Is Regulated by Changes in Gene Expression

The early phase of sporulation involves the initiation of transcription and translation of several meiotic genes (reviewed in [3]). The increase in transcription of many early meiotic genes is regulated by the master regulator of meiotic genes, *IME1* [57,58]. *IME1* is expressed by diploid cells in response to nitrogen starvation [57,59]. Among the many *IME1*-dependent genes is another master regulator of meiotic transcription, *NDT80* [60,61,62]. *NDT80* binds to specific sequences in the yeast genome known as middle sporulation elements (MSEs). The translation of Ndt80 and the subsequent upregulation of middle sporulation genes marks the transition from early sporulation to middle sporulation.

Several middle sporulation genes have their timing of expression regulated through multiple levels of transcriptional and translational control (reviewed in [63]). *SUM1* encodes a transcriptional repressor that also binds to some of the same MSEs that are bound by Ndt80 [64,65,66]. Sum1 must be removed from these MSEs prior to Ndt80 binding to promote transcription of these Ndt80 targets. Genes with promoter elements bound by Sum1 are termed middle–late sporulation genes, as their expression is delayed beyond the initial expression of Ndt80. Sum1 is removed from MSEs both through phosphorylation by Ime2, Cdk1, and Cdc7, as well as high levels of Ndt80 expression [66,67,68,69,70,71]. Phosphorylation of Sum1 weakens repressor binding to MSEs and allows Ndt80 to outcompete phosphorylated Sum1 for binding to the same DNA sequences, leading to transcriptional upregulation of affected genes. Sum1 also directly represses *NDT80* transcription, creating a feed-forward loop that also includes the increase in *IME2* transcription by Ndt80 [72].

During sporulation, there is significant post-transcriptional regulation of transcripts [73]. Sequestration of mRNA transcripts by aggregates of Rim4 may contribute to this regulation, as Rim4 aggregates sequester several transcripts in sporulation and prevent their translation until the aggregates are cleared by Ime2 phosphorylation [51,52,53]. In addition to Rim4 sequestration, the Pes4 and Mip6 RNA-binding proteins play a similar role in delaying the translation of mRNA transcripts in sporulation [74].

## 4. The Development of the Prospore Membrane Involves Initiation and Elongation

Cellularization of the spore occurs as the prospore membrane grows to surround the meiotic products (Figure 2). The fusion of post-Golgi vesicles at the meiotic outer plaque initiates prospore membrane growth and is mediated by SNAREs present on the meiotic outer plaque [6]. *ADY4* is required for a stable interaction between the meiotic outer plaque and the prospore membrane. *ADY4* was initially identified as a meiotic outer plaque component [9] and has been shown to stabilize the meiotic outer plaque through the recruitment of the lipid kinase Mss4 [75]. Furthermore, *SPO21*, which encodes another meiotic outer plaque component [7,8], appears to be important for proper prospore membrane number control. Modifications to the N-terminus of Spo21, which change positively charged residues to neutral residues, result in more than four prospore membranes initiating [75].

Many genes have been implicated in various aspects of prospore membrane development. *SPO71*, *SPO73*, and *VPS13* all appear to have roles in prospore membrane extension, as they are required for prospore membranes to become the appropriate size [27,76,77,78,79,80,81]. *SPO73* encodes a dysferlin domain-only protein that is involved in prospore membrane extension [78,79]. *SPO71* encodes a pleckstrin homology domain protein expressed specifically in sporulating cells [27,82,83]. Despite forming smaller rounded prospore membranes at the end of meiosis II, cells deficient for *SPO71* do not prematurely close their prospore membranes and do elongate prospore membranes in a timely manner [27]. *SPO71* is required to translocate Vps13 from the endosome to the prospore membrane [77]. *SPO71* and *SPO73* act cooperatively in sporulation to regulate prospore membrane morphogenesis and are together required for proper Vps13 localization to the prospore membrane [77,79].

Vps13 belongs to a family of lipid transporters that act at membrane contact sites [84,85]. In vegetatively growing cells, Vps13 localization can change depending on the nutrient conditions of cells, localizing to endosome–mitochondria junction sites on rich (glucose) media, while instead localizing to nuclear–vacuole junctions on poor (acetate) media [80,86]. *VPS13* can also facilitate lipid transfer from the Golgi to the vacuole [87,88]. Vps13 mediates lipid transfer through membrane contact sites via a large hydrophobic channel that covers the junction between two organelle membranes [86,89,90,91].

During sporulation, Vps13 localizes to prospore membranes during sporulation [76]. Localization of Vps13 to the prospore membrane is facilitated by an adaptor complex made up of Spo71 and Spo73. The Spo71-Spo73 adaptor complex also mediates the ability of Vps13 to form membrane contact sites between the ER and the prospore membrane [81]. Specifically, *VPS13* is required for the contact sites between the ER and the plasma membrane and can link ER–plasma membrane tethers along the prospore membrane. The Vps13 ER–plasma membrane contact sites are mediated by the presence of PI4P and facilitated through the Spo71-Spo73 complex [81].

Septins are also present during prospore membrane development, but do not play the same role they do during mitotic cytokinesis. The septins are present along elongating prospore membranes [92]. However, unlike in mitotically growing cells, the septins do not recruit actomyosin contractile ring components (which are not utilized during prospore membrane closure [93] (see below)). During sporulation, the septin structures are composed of slightly different multimers than in mitosis, with Spr28 and Spr3 replacing the mitotic septins Shs1 and Cdc12 [66,94]. Although Cdc12 is detectable during sporulation, it does not form higher-order structures with the other septins present in sporulation [95]. The septin genes are individually dispensable for sporulation [92,96]. However, loss of both *SPR28* and *SPR3* leads to a defect in sporulation that cannot be rescued by the overexpression of other septins [97]. *SPR28* and *SPR3* likewise cannot complement the loss of mitotic septins in mitosis, indicating that there must be some difference between the meiosis specific function of the meiotic septin complex and the mitotic septin complex [97]. It is possible that the probable loss of GTPase activity in both Spr3 and Spr28 plays some role in differentiating septin function between mitosis and meiosis [98].

Although individual septin mutants have a minimal effect on sporulation efficiency in most strain backgrounds, a role for septins during sporulation in prospore membrane elongation around the nuclear envelope was seen in the BY4743 strain background [44,45,99]. [The SK1 yeast strain background [100] is more typically used in sporulation studies, due to its ability to sporulate efficiently and somewhat synchronously]. In the BY4743 background, cells lacking *SPR3* or *SPR28* mislocalize Ady3, a component of the leading-edge protein complex (LEP; discussed below) found at the growing edge of the prospore membrane [99]. Interestingly, the septin Cdc10 appears to play a role after spore morphogenesis by marking polarization sites on the daughter spores, directing subsequent growth away from sister spores within the ascus [101,102].

Septin localization to the prospore membrane in sporulation depends on the protein phosphatase Glc7 and its sporulation-specific regulator Gip1 [31,103]. Additionally, the Glc7-Gip1 phosphatase complex is required for prospore membrane extension similar to the requirement for the Spo71-Spo73-Vps13 complex, although the two seem to operate in parallel [103]. Gip1 and Glc7 are also required for the generation of endoplasmic reticulum exit sites (ERESs), which are regenerated within the developing spores during the time of membrane extension [104]. *SPO71* is required for the localization of the sporulation-specific septin Spr28, indicating a possible link between septins and prospore membrane elongation [27].

Interestingly, the actin cytoskeleton appears to be dispensable for the development and closure of the prospore membrane. The actin cytoskeleton plays only a minor role in facilitating the transport of prospore membrane precursors and appears to be dispensable for prospore membrane initiation, extension, or closure, but is required for later spore wall deposition [93].

## 5. Removal of the Leading-Edge Protein Complex (LEP) Is Important for Prospore Membrane Closure

A protein complex that consists of at least four protein components (Don1, Ssp1, Ady3, and Irc10) is found at the leading edge of the growing prospore membrane [7,18,19,105]; this complex has been termed the leading-edge protein complex (LEP). The LEP forms a ring at the open end of each growing prospore membrane. The precise function of each protein is unknown, but it has been proposed that the LEP is responsible for exerting force to keep elongating prospore membranes open [106,107]. This protein complex is removed from the prospore membrane before it closes [106]. To date, LEP removal is the last known event to occur before the prospore membranes round up and close. Prospore membrane closure requires fusion of the open ends of the membrane to create the double lipid bilayers that fully surround the newly formed spore. Whether LEP removal is concurrent with closure or whether an additional event that promotes membrane fusion occurs after the necessary removal of the LEP is currently unknown.

Don1, the first member of the LEP to be identified, was found through a screen for meiotically expressed coiled-coil proteins and was subsequently found to form a ring-like shape at the open end of the prospore membranes [7]. This novel localization suggested that there may be a protein structure at the open end of prospore membranes. Loss of *DON1* does not result in any obvious phenotypes either in prospore membrane development or spore formation [7,25].

*SSP1* and *ADY3* were identified as members of the LEP through a Yeast Two-Hybrid screen for proteins associated with the meiotic outer plaque [18]. Though the meiotic outer plaque and the LEP are at opposite ends of the growing prospore membrane, Ssp1 and Ady3 both co-localize with Don1 at the leading edge during prospore membrane elongation, indicating that both are members of the LEP [18,19]. Loss of *SSP1* results in a severe prospore membrane formation defect in which prospore membranes collapse on the nuclear envelope, rather than maintaining a tubular structure during elongation. The C-terminal region of Ssp1 appears to be required for the degradation of Ssp1, as truncations of the protein lead to persistent expression during sporulation [106]. *ADY3* has not been shown to be integral for prospore membrane development but is required for the proper localization of Don1 to the LEP [18,19,99]. Ady3 is also required for the proper synthesis of the β-glucan and chitosan components of the spore wall [19] and proper mitochondrial segregation during sporulation [108].

The most recently discovered member of the LEP is Irc10. Identified through a visual screen of GFP-tagged ORFs expressed during meiosis, Irc10 also co-localizes with Don1. Loss of *IRC10* alone results in no obvious phenotype. However, loss of both *IRC10* and *ADY3* phenocopies loss of *SSP1*, indicating that these two members of the LEP are collectively important for the formation of the whole complex [105]. Genetic analysis utilizing loss of function alleles for the LEP members suggests a model with an order of assembly for the LEP in which Ssp1 is present on the meiotic outer plaque at prospore membrane initiation, Ady3 is recruited by Ssp1 to the LEP, then as prospore membranes elongate, both Don1 and Irc10 are recruited to the LEP [18,19,105]. After anaphase II, Ssp1 degradation requires the meiotic specific activator of the APC/C, Ama1 [109]. Ssp1 may require the Sps1 kinase for its removal from the prospore membrane for timely prospore membrane closure [110].

The sporulation MAP Kinase Smk1 also localizes to the leading edge of the prospore membrane late in meiosis II, moving from the elongating prospore membrane to briefly co-localizing with the LEP around anaphase II [25]. Interestingly, the Ssp2 activator of Smk1 [111,112,113,114,115,116] shares a similar localization as Smk1 [25], suggesting that Smk1 may be active at the LEP before prospore membrane closure. The ability of Smk1 to localize to the leading edge requires *ADY3* [25]. The localization of Smk1 without *ADY3* resembles the localization of Ssp1-YFP (which does not fully complement), which localizes ectopically in discrete areas along the prospore membrane in both wild-type and the *ady3*∆ *irc10*∆ double mutant [105]. *SMK1* also plays a role in late prospore membrane development, as *SMK1* is required for normal rounding of the prospore membrane [25]; rounding up of the prospore membrane correlates with the closure of the membrane and the cellularization of the spore [109,110]. The role *SMK1* plays in prospore membrane development may be related to its localization at the leading edge, although the exact function of Smk1 at the leading edge is still unclear.

The LEP is an important structure required for proper prospore membrane development. However, the mechanism underlying LEP localization is still unclear as is the relationship between LEP removal and prospore membrane closure.

## 6. Exit from Meiosis II: Similarities and Differences with Mitotic Exit

From the perspective of cellular events, meiosis is more complex than mitosis. Al-though both meiotic and mitotic cells must undergo DNA replication, meiosis involves two rounds of chromosome segregation: homolog segregation in meiosis I and sister chromatid segregation in meiosis II. Furthermore, the goal of meiosis is not to make a faithful copy but to create haploid gametes; this process involves shuffling the genomic content through the random segregation of homologous chromosomes plus recombination among homolog pairs. These meiotic-specific events require distinct regulatory processes (reviewed in [2]). Although some of the machinery that controls the mitotic cell cycle, like the use of cyclin-dependent kinase regulation and the spindle apparatus are similar, the details of the regulation are distinct. For example, the mitotic cyclin *CLB2* is not expressed in meiosis [117,118]. The only CDK in mitosis, Cdk1 (also known as Cdc28), is required for meiosis but acts in conjunction with the CDK-like kinase Ime2 [72,119,120,121] and utilizes some mitotic cyclins and not others; CDK regulation in meiosis has been recently reviewed [122].

In budding yeast, the mitotic division is an asymmetric event involving the formation of a bud, polarized growth, and the movement of the mitotic spindle into the newly formed bud before exit from mitosis. On the other hand, meiosis is symmetrical, with the four spores forming within the mother cell ascus. During mitosis, an actomyosin ring constricts for cytokinesis while prospore membrane closure is the cytokinetic event in meiosis. The prospore membranes round up and close after anaphase II; this process does not require the actin cytoskeleton [93].

**Figure 3:**
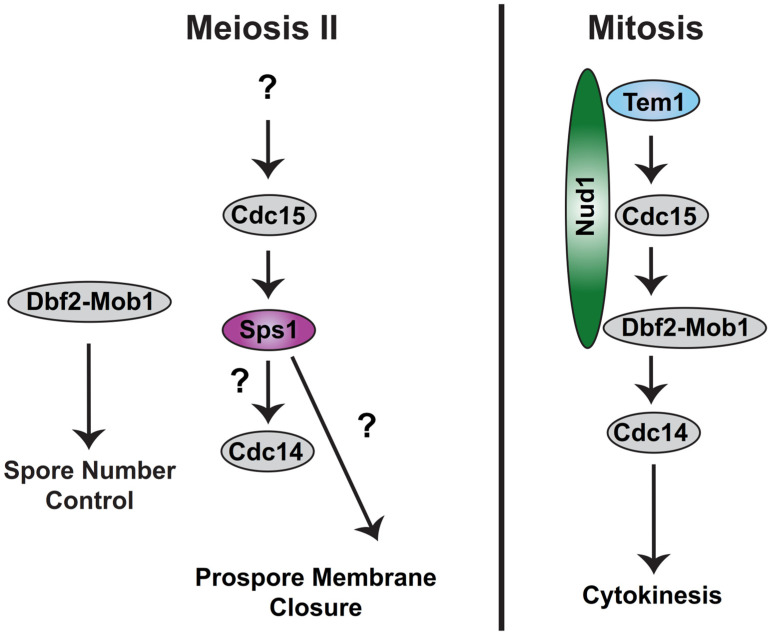
Meiotic exit pathway compared to mitotic exit. Sporulation-specific proteins are pink. Proteins used only in mitotic exit are blue or green. Proteins used in both are gray. Details in text.

This closure event marks the point when the four daughter cells have cellularized. Meiosis II happens quickly in the SK1 strain background, with anaphase II lasting about 10–20 min [53] and prospore membrane closure occurring soon after the completion of anaphase II [110]. Meiotic cytokinesis is regulated by the meiotic exit pathway [21,110,123] similar to how the end of mitosis is regulated by the mitotic exit network (sometimes known as “MEN”; reviewed in [20,124,125]) (Figure 3).

The mitotic exit network involves the activation of the Tem1 GTPase, which is activated at the spindle pole body and leads to the activation of Cdc15, a hippo-like kinase [126,127,128,129,130,131,132,133,134]. Cdc15 subsequently phosphorylates Nud1, another component of the SPB, which leads to the recruitment of the NDR-LATS kinase complex Dbf2-Mob1 [133,135,136,137]. NDR-LATS activity then leads to sustained release of the phosphatase Cdc14 from the nucleolus, which inhibits cyclin-dependent kinase (CDK) activity and promotes mitotic exit [138,139,140,141].

The Cdc14 phosphatase is an important effector of mitotic exit, reversing CDK-dependent phosphorylations and inhibiting further CDK activity so that cells return to G1 (reviewed in [142]). Cdc14 is regulated through sequestration in the nucleolus by its inhibitor Net1 until anaphase [139,143]. Two pathways control the release of Cdc14 from the nucleolus: the Cdc14 early anaphase release (FEAR) network (reviewed in [144]) and the mitotic exit network (MEN) (reviewed in [20]). The FEAR network leads to a transient release of Cdc14 in early anaphase and is partially regulated by mitotic CDKs [145,146,147]. The transient release of Cdc14 facilitates many anaphase-specific events, including nucleolar segregation and chromosome compaction [148,149,150]. Transiently released Cdc14 also promotes MEN activity by opposing the inactivation of MEN kinases by CDKs, ultimately leading to sustained Cdc14 release in late anaphase II [151,152,153]. In mitosis, Net1 is phosphorylated by the Dbf2-Mob1 kinase complex; this kinase complex is transported from the SPB to the nucleus after being phosphorylated by Cdc15 [125,154,155]. Cdc14 is also important for redistributing the GTPase Cdc42 during mitotic cytokinesis, ultimately resulting in proper cell size determination [156].

Cdc14 also affects mitotic progression by acting on an E3 ligase, the anaphase-promoting complex/cyclosome (APC/C), by dephosphorylating the late mitotic-activating subunit, Cdh1 [157]. Cdh1 activity is inhibited by Cdk1-dependent phosphorylations from the S phase until mitotic exit [158,159]. Cdh1-APC/C promotes mitotic exit through the degradation of securin, leading to the separation of sister chromatids, as well as the degradation of cyclins [160,161,162,163].

The meiotic exit pathway, which controls the timing of prospore membrane closure, requires the Cdc15 Hippo-like kinase but does not require the Tem1 GTPase or the Dbf2-Mob1 NDR/LATS kinase complex [21,164,165] (Figure 3). Instead, the Sps1 STE20 family GCKIII kinase acts downstream of Cdc15 [21]. The Nud1 SPB component, which acts as a scaffold for the mitotic exit components, is also not involved in the exit from meiosis II [165,166]. How Cdc15 is activated and whether there is a scaffold playing a role like Nud1 for the Cdc15-Sps1 pathway in meiosis is unclear. The specific role of the Cdc14 phosphatase in meiotic exit has also not been defined. Inactivating Cdc14 at metaphase II appears to block spore formation but does not seem to affect the disassembly of the meiosis II spindle [167]. The sustained release of Cdc14 from the nucleolus requires the Cdc15-Sps1 pathway [21] as well as the Hrr25 casein kinase 1 [161], although the significance of this release and the identity of which substrates require Cdc14 dephosphorylation during exit from meiosis II is currently unknown. The relationship between the Hrr25 kinase and the Cdc15-Sps1 pathway has also not been defined.

Like in mitosis, the APC/C is involved in regulating meiotic exit [109,110,168], although it utilizes a meiotic-specific activator, Ama1 [169], instead of Cdh1, as is used in mitosis. The switch in APC/C activators between mitosis and meiosis occurs because Ime2 phosphorylates many Cdk1 substrates (including Cdh1), using different phosphosites than Cdk1; these Ime2-specific phosphorylations are resistant to dephosphorylation by Cdc14, leading to the suppression of mitotic Cdk1 targets throughout meiosis [170]. During meiotic prophase, Ama1 is needed for the proteolysis of mitotic M-phase regulators like Ndd1, Cdc5, and Clb4 [171]. Ama1 activity is repressed by Mnd2, another APC/C subunit, until meiosis II [172,173]. Mnd2 is removed from the APC/C in meiosis II, at which point Ama1 targets the APC/C to degrade various proteins in sporulation including another activator of the APC/C, Cdc20 [168]. For exit from meiosis II, Ama1 (and presumably APC/C activity) is required for several processes including removal of the LEP coat, prospore membrane closure, and spindle disassembly [109,110,161,168,174,175,176].

## 7. Controlling the Timing of Meiotic Cytokinesis

The Cdc15-Sps1 pathway and the Ama1-APC/C work in parallel to regulate the proper timing of meiotic cytokinesis, as the removal of both pathways leads to continued prospore membrane elongation without closure [110]. Cells lacking *AMA1* have a partial prospore membrane closure defect, with about 30% of *ama1*∆ cells closing their prospore membranes [109,110]. Cells lacking *SPS1* also have a partial prospore membrane closure defect, with about 70% of *sps1*∆ cells rounding up and closing their prospore membranes [110]. Both *SPS1* and *AMA1* affect the timing of prospore membrane closure, as loss of each results in a delay of at least an hour without affecting the timing of prospore membrane initiation or progression through meiosis [21,110]. Strikingly, the *sps1*∆ *ama1*∆ double mutants have an additive effect: all cells form hyperelongated prospore membranes that remain open and hyperelongated [110].

While the mechanism by which *SPS1* and *AMA1* affect prospore membrane closure timing is unclear, both affect the removal and/or degradation of LEP components. Sps1 and the LEP component Ssp1 coimmunoprecipitate, indicating the two proteins complex [109]. *AMA1* is required for the normal degradation of Ssp1, as seen by immunoblotting [109]. Similarly, fluorescent microscopy indicates that both *AMA1* and *SPS1* are required for the timely removal of Don1 from the LEP [110].

## 8. Meiotic Exit Requires the Coordination of Prospore Membrane Closure and Spindle Disassembly

The closure of the prospore membrane and cellularization of the newly formed spore requires the coordination of other cellular events such as spindle disassembly, organelle segregation, nuclear envelope separation, disassembly of the meiotic outer plaque (to disassociate the nucleus from the new plasma membrane), and recycling of the nuclear pore complex through the gametogenesis uninherited nuclear compartment (known as the “GUNC”) [106,177,178,179,180,181,182]. Little is known about the coordination of these events, although the coordination of prospore membrane closure and spindle disassembly are both regulated by the Cdc15-Sps1 pathway working in conjunction with Ama1-APC/C [21,110,177].

**Figure 4:**
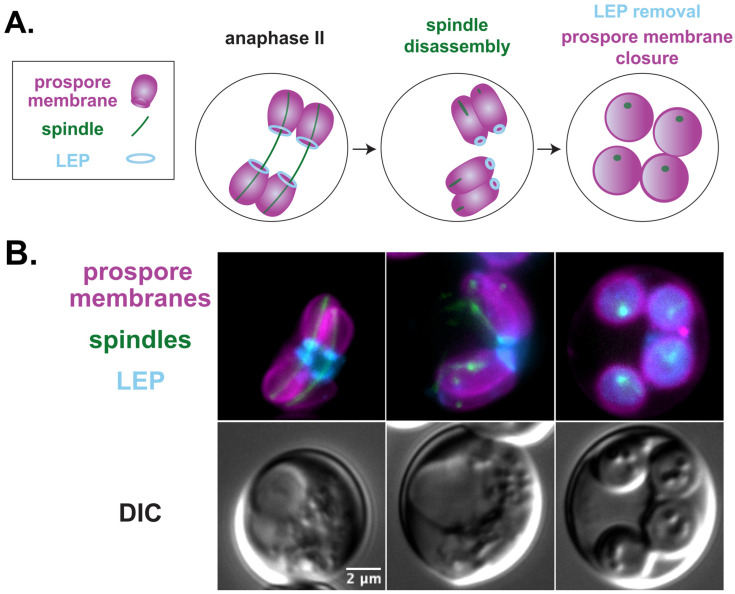
Spindle disassembly and prospore membrane closure during exit from meiosis II. (**A**) Cartoon depicting the stages of a cell during anaphase, spindle disassembly, and prospore membrane closure with time progression from left to right. Prospore membranes are labelled magenta, leading-edge protein complex (LEP) blue, and spindles green. (**B**) Prospore membrane development was examined in SK1 cells (LH1147), which contain the genomically integrated prospore membrane marker K20 (*his3::SPO20^51−91^-mKATE:HIS3* [103]) shown in magenta, leading-edge marker Don1 (*DON1-mTagBFP2::KANMX* [25]) shown in blue, and spindle marker Tub1 (alpha-tubulin; *GFP^ENVY^-TUB1+3′UTR:LEU2* [177]) shown in green. Scale bar = 2 microns. Fluorescent images are maximum intensity projections of 3 µm z-stacks merged taken on a Zeiss Axioskop Mot2 wide-field microscope, using a 100× objective (NA 1.45).

The first indication that both the Cdc15-Sps1 and Ama1-APC/C pathways were involved in coordinating spindle disassembly and prospore membrane closure was that all mutants in these pathways produce hyperelongated prospore membranes and have meiosis II spindle disassembly defects. In addition to the hyperelongated prospore membranes seen in cells lacking *CDC15*, *SPS1*, or *AMA1*, these mutant cells also had persistent tubulin fragments that remained after anaphase II. This observation led to the question of whether the delay in the closure of the prospore membrane seen in these mutants was due to a problem with meiosis II spindle disassembly. In wild-type cells, the spindle disassembles before the prospore membrane closes (Figure 4).

Interestingly, disassembly of the meiosis II spindle in cells with hyperelongated prospore membranes does not cause prospore membrane closure, consistent with the idea that these two events are regulated separately [177]. In wild-type cells, the addition of a benomyl–nocodozole cocktail that depolymerized microtubules after anaphase II resulted in normal prospore membrane closure shortly after the spindles were disassembled. In contrast, although the addition of the benomyl–nocodozole cocktail to cells lacking either *SPS1* and *AMA1* completely disassembled the spindles, the prospore membranes continued to elongate for up to an hour after the addition of the benomyl–nocodozole cocktail, indicating that the hyperelongated prospore membranes are not due to persistent intact microtubule fragments seen in these mutants.

Although both prospore membrane closure and spindle disassembly are regulated by Cdc15-Sps1 and Ama1-APC/C [21,109,110,164,165,177], these pathways likely act on distinct downstream targets for each process and thus, the activity of these pathways provides the coordination of the two events. Potential targets for Cdc15-Sps1 and Ama1-APC/C for spindle disassembly include the microtubule-binding proteins Bim1, Cin8, and Ase1, whose localization in meiosis II is impacted by the loss of either *SPS1* or *AMA1* [177]. Ssp1 and other components of the LEP may be important targets for prospore membrane closure [106,109,110]. Cdc14, an effector for mitotic exit, may also play a role in meiotic exit, as the Cdc15-Sps1 pathway and the Hrr25 casein kinase affect Cdc14 release from the nucleolus in anaphase II [21,167]. However, the specific requirement for Cdc14 release in meiosis II has not been determined.

## 9. Conclusions

Progress has been made in understanding the mechanisms underlying meiotic cytokinesis in *S. cerevisiae*, but there are still important areas to explore. Sporulation involves a diverse array of cell biological processes, including the creation of a new membrane, microtubule spindle assembly and disassembly, and organellar segregation. Such processes are important in all eukaryotes and operate across a variety of cellular contexts, so insights obtained from the study of sporulation can help inform our understanding of fundamental cell biological processes more broadly.

Furthermore, a better understanding of this cellularization process can inform our understanding of other systems. Although prospore membrane closure occurs using mechanisms distinct from typical animal cell cytokinesis, atypical cellularization events occur in both mitosis and meiosis across species from insects to mammals [183]. The fission yeast *Schizosaccharomyces pombe* (which is thought to have diverged from the budding yeast *S. cerevisiae* 350 million years ago [184]) also produces spores though the production of a forespore membrane that grows from a meiotically modified SPB [185,186]. However, unlike the budding yeast, the closure of the forespore membrane in *S. pombe* requires a meiotic actin ring [187]. The cellularization of the nuclei in early *Drosophila* development is one of the better-studied examples of atypical cytokinesis and involves extensive membrane remodeling and growth to surround the mitotic nuclei (reviewed in [188]). In mammalian females, the germline undergoes incomplete cytokinesis through intercellular bridges, resulting in a syncytium structure [189]. Better understanding of prospore membrane closure as the meiotic cytokinetic event may inform our understanding of other atypical cytokinetic events. Thus, the further examination of meiotic cytokinesis using the powerful tools available in *S. cerevisiae* is likely to have impacts well beyond the creation of the spore in budding yeast.

## Data Availability

No new data were created or analyzed in this study. Data sharing is not applicable to this article.
